# Maturation-, age-, and sex-specific anthropometric and physical fitness percentiles of German elite young athletes

**DOI:** 10.1371/journal.pone.0237423

**Published:** 2020-08-13

**Authors:** Melanie Lesinski, Alina Schmelcher, Michael Herz, Christian Puta, Holger Gabriel, Adamantios Arampatzis, Gunnar Laube, Dirk Büsch, Urs Granacher

**Affiliations:** 1 Division of Training and Movement Sciences, University of Potsdam, Research Focus Cognition Sciences, Potsdam, Germany; 2 Department of Sports Medicine and Health Promotion, Friedrich Schiller University, Jena, Germany; 3 Department of Training and Movement Sciences, Humboldt‐Universität zu Berlin, Berlin, Germany; 4 Berlin School of Movement Science, Berlin, Germany; 5 Institute of Sports Science, Carl von Ossietzky University of Oldenburg, Oldenburg, Germany; Universidade Federal de Mato Grosso do Sul, BRAZIL

## Abstract

The aim of this study was to establish maturation-, age-, and sex-specific anthropometric and physical fitness percentile reference values of young elite athletes from various sports. Anthropometric (i.e., standing and sitting body height, body mass, body mass index) and physical fitness (i.e., countermovement jump, drop jump, change-of-direction speed [i.e., T-test], trunk muscle endurance [i.e., ventral Bourban test], dynamic lower limbs balance [i.e., Y-balance test], hand grip strength) of 703 male and female elite young athletes aged 8–18 years were collected to aggregate reference values according to maturation, age, and sex. Findings indicate that body height and mass were significantly higher (p<0.001; 0.95≤d≤1.74) in more compared to less mature young athletes as well as with increasing chronological age (p<0.05; 0.66≤d≤3.13). Furthermore, male young athletes were significantly taller and heavier compared to their female counterparts (p<0.001; 0.34≤d≤0.50). In terms of physical fitness, post-pubertal athletes showed better countermovement jump, drop jump, change-of-direction, and handgrip strength performances (p<0.001; 1.57≤d≤8.72) compared to pubertal athletes. Further, countermovement jump, drop jump, change-of-direction, and handgrip strength performances increased with increasing chronological age (p<0.05; 0.29≤d≤4.13). In addition, male athletes outperformed their female counterpart in the countermovement jump, drop jump, change-of-direction, and handgrip strength (p<0.05; 0.17≤d≤0.76). Significant age by sex interactions indicate that sex-specific differences were even more pronounced with increasing age. Conclusively, body height, body mass, and physical fitness increased with increasing maturational status and chronological age. Sex-specific differences appear to be larger as youth grow older. Practitioners can use the percentile values as approximate benchmarks for talent identification and development.

## Introduction

There are primarily two pathways which are pursued to develop a gifted young child into a talented elite athlete. These are early specialization and diversification [1]. While both pathways have proven to be successful in developing high performance athletes, more recent evidence has shown that there is an increased risk with early specialization to sustain acute and/or overuse injuries which may ultimately lead to drop out from organized sports [2, 3]. Diversification on the other hand has proven to be particularly successful with cgs (centimeters, grams, seconds) sports in terms of developing successful elite athletes [4]. A premise of the diversification approach is to lay a foundation of physical fitness before developing sport-specific performance [5]. In other words, fitness development precedes sport specialization. Accordingly, reference values are needed for this first step of the diversification approach to evaluate physical fitness levels of youth athletes, irrespective of the sport they practice. To pursue this promising approach, reference data are needed of various physical fitness tests to support coaches and athletes with talent identification, selection, and development [6]. This will help to better monitor and guide the development of talented youth athletes on their early diversification pathway. For the general youth population, studies exist with large cohorts that provide age- and sex-specific percentile norm values for different anthropometric and physical fitness outcomes (e.g., hand grip strength, 1-kg ball push, standing long jump, 50-m sprint, shuttle run test) [7–9]. These studies suggest that anthropometry and physical fitness develop with increasing age in a sex-specific non-linear fashion. However, these data sets cannot be utilized with young athletes because by definition, talented youth are equal to or above the 90^th^ percentile of the respective general population [10, 11]. Superior performance of young sporting talents is due to both, nature (i.e., genes) but also nurture (e.g., regular training over several years) [12]. Accordingly, it is timely and imperative to establish cohort specific reference values that can be used for talent identification, selection, and development. However, previous studies with young athletes reported only age-, sex-, and/or sport-specific mean values for different physical fitness outcomes [13–15]. For instance, Opstoel et al. [14] determined mean values of 620 children aged 9 to 11 who participated in at least one specific sport (i.e., in total 25 different sports) for several physical fitness outcomes (e.g., hand grip strength, countermovement jump [CMJ], standing long jump, shuttle run test). Yet, there is currently no study available that has established maturation-specific anthropometric and physical fitness percentiles of elite young athletes. Of note, maturation is a non-linear process, which is why there is often a discrepancy between chronological age and maturation among young athletes [16–18]. This is a major challenge in youth sport where competitions are mainly regulated by chronological age-groups.

To the authors’ knowledge, there are no studies available that provide maturation-, age-, and sex-specific anthropometric and physical fitness percentiles for young athletes. Therefore, the purpose of this cross-sectional study was to present and discuss maturation, age, and sex-specific anthropometric (e.g., body height, body mass) and physical fitness (e.g., CMJ, drop jump [DJ], change-of-direction [CoD] speed) percentile reference values of young elite athletes from various sports. With reference to the relevant literature [16, 19, 20], we hypothesized that body height, body mass, and physical fitness increase with age and maturation in a sex-specific but non-linear fashion.

## Materials and methods

### Participants

A convenience sample of 703 male (♂ = 420) and female (♀ = 283) young elite athletes aged 8–18 years who originally participated in a large research project entitled “Resistance Training in Young Athletes” (https://www.uni-potsdam.de/kraftprojekt/english.php) was used to aggregate anthropometric and physical fitness reference values. Athletes were from 18 different sports including soccer (41 ♀/ 49 ♂), volleyball (24 ♀/24 ♂), basketball (10 ♀/29 ♂), handball (29 ♀/84 ♂), judo (36 ♀/53 ♂), wrestling (3 ♀/6 ♂), boxing (3 ♀/7 ♂), canoeing (21 ♀/42 ♂), rowing (21 ♀/0 ♂), ski jumping (0 ♀/18 ♂), nordic combination (5 ♀/15 ♂), speed skating (13 ♀/8 ♂), swimming (37 ♀/26 ♂), weight lifting (3 ♀/7 ♂), badminton (13 ♀/8 ♂), gymnastics (0 ♀/18 ♂), athletics (13 ♀/11 ♂), and modern pentathlon (12 ♀/14 ♂). Participants were recruited from German elite sport schools and followed a training regime consisting of regular physical education together with their sport-specific training and competitions. On average, participating athletes practiced their sport between 2 and 12 years. All athletes were enrolled at elite sport schools and performed a minimum of 10 hours of training per week. Each participant was coded for his/her maturity status, age, and sex. Maturity was determined by calculating the time from peak-height-velocity (PHV) according to the regression equations of Mirwald et al. [21] for boys:

Maturity offset = -9.236 + (0.0002708 * leg length * sitting height)—(0.001663 * age * leg length) + (0.007216 * age* sitting height) + (0.02292 * weight by height ratio) and girls:

Maturity offset = -9.376 + (0.0001882 * leg length * sitting height) + (0.0022 * age * leg length) + (0.005841·age * sitting height)—(0.002658·age * weight) + (0.07693 * weight by height ratio).

In accordance with Faigenbaum et al. [22], maturity was classified as pre-pubertal (i.e., < -1 year before PHV), pubertal (i.e., ±1 years around PHV), and post-pubertal (i.e., > 1 year after PHV). Prior to the start of the study, all participants were informed about potential risks and benefits of the study and athletes as well as their legal guardians provided their written informed consent. The protocol was approved by local ethical commissions (University of Potsdam: submission No. 5/2014; Charité Berlin: EA2/076/15; Friedrich-Schiller-University Jena: 458510/15).

### Testing procedures

Baseline data of anthropometric and physical fitness tests were used from intervention studies of a larger research project to aggregate reference values. All anthropometric and physical fitness tests were performed under strictly standardized conditions. In these studies, baseline testing always started with the assessment of anthropometrics (i.e., standing and sitting height, body mass). Tests were always conducted in the morning before fitness testing. According to Mirwald et al. [21], standing and sitting height were measured to the nearest mm. Two measurements were taken for each anthropometric variable and averaged for analysis. A third measurement was required if the first two differed by more than 4 mm for standing or sitting height. Prior to physical fitness testing, a standardized warm-up protocol (i.e., ten minutes of jumping, running and agility/change-of-direction drills) was performed. The physical fitness test battery included the assessment of vertical jump performance (i.e., DJ, CMJ), CoD speed (i.e., T-test), dynamic balance of the lower extremities (i.e., Y balance-test), trunk muscle endurance (i.e., ventral Bourban-test), and hand grip strength. Participants were familiarized with all physical fitness tests prior to data assessment. Hand and leg dominance were determined using the lateral preference inventory [23].

#### Assessment of hand grip strength

Grip strength of the dominant hand was measured using a hand-held dynamometer (Jamar Plus, Performance Health, Warrenville, IL, USA) which showed good test-retest (ICC > 0.80) and inter-rater reliability (ICC > 0.97) [24]. During testing, participants were seated upright, elbows by the side of the body and flexed at an angle of 90°. Participants were instructed to press the dynamometer grip as forcefully as possible for 5 s while maintaining their position (i.e., no additional movements from upper or lower body). Three trials were conducted and the best trial was used for further analysis.

#### Assessment of jump performance

CMJ and DJ performances were measured using an optoelectric cell system (Optojump, Microgate, Bolzano, Italy). Excellent test–retest reliability (intraclass correlation coefficient [ICC]) was previously reported for the estimation of vertical jump height using the Optojump photocell system (ICC = 0.98) [25]. For CMJ, athletes stood in an upright erect standing position, feet shoulder-width apart, and hands akimbo. CMJs were initiated with a countermovement which was immediately followed by a concentric explosive upward movement. For DJ, participants stood in an upright erect standing position on a 40 cm box, feet shoulder-width apart, and hands akimbo. Participants were asked to step off the box with their dominant leg, drop down to land evenly on both feet on the ground, keep ground contact time short, and jump-off the ground with a double-leg vertical jump at maximal effort. All participants jumped with shoes as well as were instructed to jump as high as possible (CMJ, DJ) and to keep ground contact as short as possible (DJ). Three trials were conducted for each jump test. The best trial in terms of maximal jump height (CMJ, DJ) was taken for further analysis. Furthermore, participants’ performance index was calculated using the following formula: DJ performance index = drop jump height [m] / contact time [s]. The best trial in terms of maximal DJ performance index was taken for further analysis.

#### Assessment of trunk muscle endurance

Endurance of the trunk muscles was assessed using the ventral Bourban-test. The test has previously proven to be valid as well as reliable with an ICC of 0.87 [26]. During test performance, athletes are in plank position, elbows shoulder-with apart, forearms flat on the ground and legs extended. A reference rod of the alignment device touched the athlete´s lower back at the iliac crests. In this position, athletes were asked to lift their feet (2–5 cm) alternately (i.e., 1 s per foot) for as long as possible according to the beat of a metronome. If athletes lost contact with the reference rod for longer than 2–4 seconds, they received a warning from the test instructor. Test time until failure was recorded using a stopwatch and taken as dependent variable. Alternatively, test time to the third warning was used for further analysis.

#### Assessment of change-of-direction speed

Change-of-direction speed (CoD) was assessed using the T-test [27], which showed high test-retest reliability with an ICC of 0.98 [27]. Athletes had to complete a course, set up as a “T” using four cones, in the shortest possible time. For this purpose, they sprinted forward, performed sidesteps and ran backwards. The athletes started without a start signal and sprint time was measured using a double-light electronic gate system (WITTY; Microgate Srl, Bolzano, Italy). Following a submaximal test trial, the fastest out of two trials was taken for further analysis.

#### Assessment of dynamic balance

The lower quarter Y balance-test was used to assess dynamic balance [28]. According to Plisky et al. [28], ICC values for the three different movement directions ranged between 0.89 and 0.93 and showed high test-retest reliability. Athletes were barefooted and positioned in single leg stance on the Y-Balance-Test-Kit (Move2Perform, Evansville, IN, USA). They were asked to reach with the contralateral leg as far as possible into three different movement directions (i.e., ventral, posteromedial, posterolateral). Athletes always started the test while they stood on the right leg. With the left leg, participants had to reach three times in one direction before switching sides and directions. For familiarization purposes, all athletes completed three trials per leg and per movement direction before the tests started. The best performance (furthest reach) in each direction was used for further analysis. According to Filipa et al. [29], a composite score was calculated according to the equation: composite score = [(maximum anterior reach distance + maximum posteromedial reach distance + maximum posterolateral reach distance)/(leg length × 3)] × 100 and taken as dependent variable for further analysis. Of note, leg length was assessed by measuring the distance between the anterior superior iliac spine and the most distal aspect of the medial malleolus while the athlete lies in supine position.

### Statistical analyses

Data are mean values and standard deviations (SDs) with 95% confidence intervals for anthropometrics and physical fitness. After data were tested and confirmed for normal distribution (i.e., Shapiro Wilk test), an univariate ANOVA was applied with the factors sex, maturity status, and age as between subject comparators. Bonferroni corrected post-hoc tests were computed for multiple comparisons to determine outcomes according to maturation and age. The level of significance was set at p < 0.05 for each comparison. In addition, the classification of effect sizes was determined by calculating Cohen’s d from partial eta-squared. Effect sizes constitute a means to determine whether a difference is a difference of practical concern. According to Cohen [30], effect sizes can be classified as small (d < 0.5), medium (0.5 ≤ d < 0.8), or large (d ≥ 0.8). Percentile analyses were computed separately for boys and girls according to maturation and age. The 20^th^, 40^th^, 50^th^, 60^th^, and 80^th^ percentiles were calculated. Due to the limited overall data pool of elite young athletes [31] and in accordance with other authors [32, 33], anthropometric and physical fitness differences as well as percentile reference values were only calculated if 30 participants were available within a subgroup. All analyses were conducted using IBM SPSS Statistics for Windows, Version 26.0 (IBM Corp., Armonk, NY, USA).

## Results

### Anthropometry and physical fitness differences by maturity status, age, and sex

Tables 1 and 2 contain sex-specific anthropometric and physical fitness values according to chronological age (Table 1) and maturity status (Table 2).

**Table 1:** Anthropometric and physical fitness differences according to chronological age and sex in young athletes.

	chronological age								main/interaction effects		
	12		13		14		15		p-value (d)		
	boys	girls	boys	girls	boys	girls	boys	girls	age	sex	age x sex
	mean ± SD	mean ± SD	mean ± SD	mean ± SD	mean ± SD	mean ± SD	mean ± SD	mean ± SD	p (d)	p (d)	p (d)
	(95% CI)	(95% CI)	(95% CI)	(95% CI)	(95% CI)	(95% CI)	(95% CI)	(95% CI)			
	n = 55	n = 45	n = 91	n = 61	n = 69	n = 56	n = 76	n = 52			
ANTHROPOMETRY											
standing height [cm]	161.3 ± 11.7	162.2 ± 10.0	168.4 ± 10.7	165.7 ± 7.5	170.4 ± 10.0	166.9 ± 8.2	179.0 ± 9.9	172.2 ± 8.3	<0.001	<0.001	0.01
	(158.7–163.9)	(159.3–165.0)	(166.5–170.5)	(163.3–168.2)	(168.1–172.7)	(164.3–169.4)	(176.8–181.2)	(168.5–173.8)			-0.3
	n = 54	n = 45	n = 91	n = 59	n = 68	n = 56	n = 76	n = 52	-0.94	-0.34	
sitting height [cm]	83.2 ± 6.2	84.5 ± 4.9	86.7 ± 5.2	86.8 ± 4.1	87.4 ± 5.5	87.6 ± 3.9	91.3 ± 5.8	89.3 ± 3.8	<0.001	0.872	0.104
	(81.9–84.5.6)	(83.1–86.1)	(85.7–87.8)	(85.5–88.1)	(86.1–88.7)	(86.3–89.0)	(90.1–92.5)	(87.9–90.7)		-0.01	-0.3
	n = 55	n = 44	n = 90	n = 58	n = 60	n = 54	n = 67	n = 51	-0.86		
BMI [kg/m^2^]	19.0 ± 3.0	19.3 ± 2.5	19.4 ± 2.9	20.2 ± 2.5	20.0 ± 2.7	20.9 ± 2.4	21.6 ± 3.1	20.9 ± 3.7	<0.001	0.538	0.035
	(18.1–19.9)	(18.4–20.3)	(18.8–20.2)	(19.4–21.1)	(19.2–20.8)	(20.2–21.8)	(20.9–22.4)	(19.9–21.8)		-0.06	-0.27
	n = 53	n = 45	n = 88	n = 58	n = 64	n = 54	n = 69	n = 50	-0.5		
body mass [kg]	50.2 ± 13.0	51.4 ± 10.7	55.7 ± 13.2	55.6 ± 9.4	58.4 ± 12.0	58.6 ± 8.4	69.5 ± 14.2	62.3 ± 8.5	<0.001	0.114	0.009
	(46.8–53.5)	(47.7–55.0)	(53.1–58.3)	(52.4–58.8)	(55.3–61.4)	(55.2–61.9)	(66.1–72.9)	(58.7–65.8)		-0.15	-0.31
	n = 53	n = 44	n = 88	n = 60	n = 64	n = 54	n = 69	n = 49	-0.88		
PHYSICAL FITNESS											
CMJ height [cm]	26.0 ± 5.6	23.6 ± 3.4	27.6 ± 4.3	25.0 ± 4.4	30.4 ± 7.2	26.5 ± 4.3	36.2 ± 8.8	27.4 ± 5.5	<0.001	<0.001	<0.001
	(24.2–25.3)	(21.9–25.3)	(26.4–28.8)	(23.5–26.5)	(28.8–31.9)	(25.0–28.0)	(34.7–37.6)	(25.8–29.1)			
	n = 43	n = 42	n = 86	n = 53	n = 51	n = 55	n = 61	n = 47	-0.89	-0.76	-0.45
DJ height [cm]	22.1 ± 5.4	22.5 ± 5.0	23.8 ± 4.9	22.4 ± 5.0	26.7 ± 6.6	23.4 ± 4.0	31.0 ± 7.3	24.8 ± 5.0	<0.001	<0.001	<0.001
	(20.5–23.8)	(20.9–24.2)	(22.6–24.9)	(21.0–23.9)	(25.1–28.2)	(21.9–24,8)	(29.6–32.3)	(23.3–26.3)			
	n = 43	n = 42	n = 86	n = 52	n = 51	n = 55	n = 62	n = 49	-0.77	-0.47	-0.43
DJ ground	251±101	242±53	232±61	215±42	230±67	209±29	204±35	211±35	<0.001	0.078	0.287
contact time [ms]	(220–283)	(226–258)	(218–245)	(203–227)	(218–245)	(201–217)	(195–249)	(201–221)		-0.01	-0.009
	n = 43	n = 42	n = 86	n = 52	n = 51	n = 55	n = 62	n = 49	-0.05		
DJ performance	0.97 ± 0.39	0.96 ± 0.28	1.07 ± 0.29	1.08 ± 0.30	1.27 ± 0.40	1.14 ± 0.26	1.59 ± 0.50	1.21 ± 0.36	<0.001	<0.001	<0.001
index [m/s]	(0.9–1.1)	(0.9–1.1)	(1.0–1.1)	(1.0–1.2)	(1.2–1.4)	(1.0–1.2)	(1.5–1.7)	(1.1–1.3)			
	n = 43	n = 42	n = 86	n = 52	n = 51	n = 55	n = 62	n = 49	-0.88	-0.35	-0.44
T-test [s]	11.71 ± 1.08	12.14 ± 0.71	10.98 ± 0.72	11.73 ± 0.94	10.82 ± 0.91	11.23 ± 0.96	10.19 ± 0.75	10.92 ± 0.93	<0.001	<0.001	0.309
	(11.4–12.0)	(11.9–12.4)	(10.8–11.2)	(11.5–12.0)	(10.6–11.1)	(11.0–11.5)	(10.0–10.4)	(10.7–11.2)			-0.19
	n = 34	n = 42	n = 75	n = 52	n = 51	n = 51	n = 62	n = 48	-1.08	-0.66	
Y-balance [%]	103.9 ± 8.9	101.1 ± 4.6	101.3 ± 6.9	102.6 ± 6.9	103.7 ± 11.2	104.3 ± 6.1	104.9 ± 10.0	101.9 ± 8.1	0.5	0.286	0.263
	(101.0–106.9)	(97.8–104.3)	(98.6–104.0)	(99.9–105.3)	(100.9–106.4)	(101.3–106.7)	(102.5–107.3)	(99.4–104.4)	-0.19	-0.13	-0.24
	n = 30	n = 25	n = 36	n = 36	n = 34	n = 36	n = 45	n = 42			
Bourban-test [s]	112.6 ± 50.1	102.2 ± 61.5	162.4 ± 178.2	141.0 ± 163.4	152.4 ± 108.1	163.0 ± 307.5	120.9 ± 44.2	103.2 ± 38.1	<0.001	0.515	0.869
	(65.9–159.4)	(56.5–147.8)	(129.1–195.6)	(100.3–181.7)	(110.5–194.3)	(121.4–204.5)	(82.5–159.2)	(60.0–146.4)		-0.06	-0.08
	n = 41	n = 42	n = 80	n = 53	n = 51	n = 52	n = 61	n = 48	-0.29		
Hand grip	29.2 ± 8.0	26.2 ± 4.6	33.1 ± 8.7	30.5 ± 5.2	33.5 ± 9.3	32.0 ± 4.5	41.3 ± 10.5	32.7 ± 5.9	<0.001	<0.001	0.02
strength [kg]	(26.8–31.6)	(23.8–28.5)	(31.3–34.9)	(28.3–32.7)	(31.3–35.7)	(29.5–34.4)	(39.1–43.5)	(29.9–35.4)			-0.33
	n = 40	n = 40	n = 74	n = 47	n = 47	n = 39	n = 48	n = 30	-0.8	-0.5	

Data were only calculated if at least 30 participants were available within a subgroup. For the subgroups of 8, 9, 10, 11, 16, 17, and 18 years

old young athletes there were less than 30 participants and, thus, data were not calculated and reported.

BMI = body mass index, CMJ = countermovement jump, DJ = drop jump, SD = standard deviation.

**Table 2:** Anthropometric and physical fitness differences according to maturity status and sex in young athletes.

	biological age						main/interaction effects		
	pre-pubertal		pubertal		post-pubertal		p-value (d)		
	(maturity offset: -1.98±0.71)		(maturity offset: 0.04±0.55)		(maturity offset: 2.57±1.07)				
	boys	girls	boys	girls	boys	girls	age	sex	age x sex
	mean ± SD	mean ± SD	mean ± SD	mean ± SD	mean ± SD	mean ± SD	p (d)	p (d)	p (d)
	(95% CI)	(95% CI)	(95% CI)	(95% CI)	(95% CI)	(95% CI)			
	n = 78	n = 4	n = 162	n = 54	n = 149	n = 216			
ANTHROPOMETRY									
standing height [cm]	150.9 ± 10.5	142.3 ± 9.5	167.9 ± 8.7	155.8 ± 7.3	183.5 ± 9.6	169.7 ± 7.9			
	(148.9–153.0)	(132.3–152.3)	(166.5–169.3)	(153.4–158.2)	(182.1–184.9)	(168.5–170.9)	<0.001	<0.001	0.398
	n = 77	n = 3	n = 162	n = 53	n = 149	n = 216	-1.57	-0.5	-0.11
sitting height [cm]	78.1 ± 4.8	76.1 ± 2.5	86.0 ± 4.4	81.9 ± 4.6	93.3 ± 5.7	88.4 ± 4.0			
	(77.1–79.1)	(71.5–80.7)	(85.3–86.7)	(80.6–83.1)	(92.5–94.1)	(87.8–89.0)	<0.001	<0.001	0.389
	n = 78	n = 3	n = 162	n = 54	n = 141	n = 211	-1.44	-0.34	-0.11
BMI [kg/m^2^]	17.5 ± 2.1	16.0 ± 0.1	19.5 ± 2.7	18.5 ± 2.0	22.2 ± 3.1	21.1 ± 2.5			
	(17.0–18.0)	(15.7–16.3)	(19.1–19.9)	(17.9–19.0)	(21.7–22.8)	(20.7–21.4)	0.004	0.554	0.929
	n = 76	n = 3	n = 156	n = 53	n = 134	n = 204	-0.26	-0.05	-0.03
body mass [kg]	40.3 ± 8.7	32.6 ± 4.4	55.2 ± 11.5	45.0 ± 7.4	74.8 ± 14.6	60.9 ± 9.9			
	(38.3–42.2)	(21.6–43.5)	(53.4–57.1)	(43.0–47.1)	(72.3–77.4)	(59.5–62.2)	<0.001	<0.001	0.066
	n = 76	n = 4	n = 156	n = 54	n = 132	n = 203	-1.42	-0.39	-0.19
PHYSICAL FITNESS									
CMJ height [cm]	25.1 ± 4.2	NA	28.5 ± 6.1	24.3 ± 3.8	37.7 ± 8.8	26.6 ± 5.0			
	(23.6–26.6)	n = 1	(27.6–29.5)	(22.5–26.0)	(36.7–38.8)	(25.7–27.4)	<0.001	0.041	<0.001
	n = 64		n = 151	n = 48	n = 131	n = 209	-0.77	-0.17	-0.48
DJ height [cm]	21.4 ± 4.3	NA	24.6 ± 6.4	22.7 ± 5.2	31.1 ± 6.9	24.0 ± 4.9			
	(19.9–22.7)	n = 1	(23.6–25.5)	(21.1–24.3)	(30.1–32.1)	(23.3–24.8)	<0.001	0.357	0
	n = 63		n = 151	n = 48	n = 135	n = 209	-0.57	-0.08	-0.39
DJ ground contact time [ms]	213 ± 55	NA	237 ± 78	244 ± 66	207 ± 44	213 ± 38	<0.001		
	(199–227)	n = 1	(224–249)	(224–263)	(199–215)	(208–219)		0.83	0.845
	n = 63		n = 151	n = 48	n = 135	n = 209	-0.05	-0.02	-0.001
DJ performance index [m/s]	1.1 ± 0.3	NA	1.1 ± 0.4	1.0 ± 0.3	1.6 ± 0.5	1.2 ± 0.3			
	(1.0–1.1)	n = 1	(1.1–1.2)	(0.9–1.1)	(1.5–1.6)	(1.1–1.2)	<0.001	0.482	0.002
	n = 63		n = 151	n = 48	n = 135	n = 209	-0.63	-0.06	-0.29
T-test [s]	12.0 ± 0.8	NA	11.1 ± 0.9	12.4 ± 1.0	10.1 ± 0.7	11.3 ± 1.0			
	(11.8–15.2)	n = 1	(10.9–11.2)	(12.2–12.7)	(9.9–10.2)	(11.1–11.4)	<0.001	<0.001	0.504
	n = 58		n = 131	n = 47	n = 136	n = 205	-1.04	-0.37	-0.1
Y-balance test [%]	103.7 ± 5.6	108.2 ± 13.7	103.5 ± 10.8	107.3 ± 4.2	105.8 ± 10.0	102.6 ± 7.0			
	(101.1–106.4)	(98.6–117.8)	(101.4–105.5)	(103.1–111.4)	(104.0–107.6)	(101.2–104.0)	0.567	0.378	0.014
	n = 39	n = 3	n = 64	n = 16	n = 88	n = 148	-0.11	-0.09	-0.31
Bourban-test [s]	338.1 ± 481.2	NA	153.1 ± 150.0	133.7 ± 70.0	125.4 ± 56.8	130.9 ± 180.1			
	(286.9–389.2)	n = 1	(119.3–187.0)	(74.9–192.4)	(90.4–160.3)	(103.0–159.0)	0.354	0.429	0.64
	n = 62		n = 142	n = 47	n = 132	n = 204	-0.12	-0.07	-0.08
Hand grip strength [kg]	23.1 ± 4.5	NA	32.2 ± 7.5	23.8 ± 4.3	43.9 ± 10.0	31.5 ± 5.3			
	(21.2–24.9)	n = 1	(31.0–33.4)	(21.5–26.1)	(42.5–45.3)	(30.4–32.6)	0.000	0.001	0.026
	n = 55		n = 130	n = 35	n = 99	n = 153	-1.16	-0.3	-0.25

Maturity was determined by calculating the time from peak-height-velocity (PHV) according to the equations as provided by Mirwald et al., [21]. Maturity was classified as pre-pubertal (i.e., > 1 year before PHV), pubertal (i.e., ±1 year around PHV), and post-pubertal (i.e., > 1 year after PHV).

BMI = body mass index, CMJ = countermovement jump, DJ = drop jump; NA = not applicable; SD = standard deviation.

#### Effects of chronological age

Significant main effects of chronological age were found for all anthropometric and physical fitness test values (p < 0.05; 0.29 ≤ d ≤ 1.08), except for the Y-balance test (p > 0.05; d = 0.19) (Table 1). Post-hoc analyses indicated significantly higher body height and mass with increasing age (p < 0.05; 0.66 ≤ d ≤ 3.13), except for 13 and 14 years old athletes (p > 0.05; 0.14 ≤ d ≤ 0.25). Furthermore, post-hoc analyses indicated significantly better hand grip strength, CMJ, DJ, and CoD performances with increasing age (p < 0.05; 0.40 ≤ d ≤ 4.27). Notably, jump performance did not increase considerably between 12 and 13 year old athletes (p > 0.05; 0.18 ≤ d ≤ 0.39). Furthermore, hand grip strength did not improve considerably in athletes aged 13 and 14 years (p > 0.05; 0.09 ≤ d ≤ 0.25).

#### Effects of maturity status

Significant main effects of maturity were found for all anthropometric and physical fitness tests (p < 0.01; 0.26 ≤ d ≤ 1.57), except for the Y-balance and the Bourban test (p > 0.05; 0.11 ≤ d ≤ 0.12) (Table 2). Post-hoc analyses indicated that body height and mass were significantly higher (p < 0.001; 0.95 ≤ d ≤ 1.85) in more matured young athletes (i.e., pre-pubertal < pubertal < post-pubertal). Further, post-pubertal compared to pubertal athletes showed significantly better performances in jump (i.e., CMJ height, DJ height, DJ performance index, DJ ground contact time) and CoD tests (p < 0.001; 1.57 ≤ d ≤ 3.13).

#### Effects of sex

Furthermore, significant main effects of sex were found for anthropometric and physical fitness tests. More precisely, male young athletes were significantly taller and heavier compared with female young athletes (p < 0.001; 0.34 ≤ d ≤ 0.50). Furthermore, males outperformed females in CMJ, DJ, CoD performances and hand grip strength (p < 0.05; 0.17 ≤ d ≤ 0.76) (Tables 1 and 2).

#### Interaction effects of the factors maturity, age, and sex

Our analyses showed significant sex by maturity interactions for almost all physical fitness tests (p < 0.05; 0.25 ≤ d ≤ 0.48), except for the T-test and the Bourban-test (p > 0.05; 0.08 ≤ d ≤ 0.10) (Table 2). Post-hoc analyses indicated more pronounced sex-specific differences (i.e., better CMJ, DJ, DJ performance index, and hand grip strength in boys compared to girls) in post-pubertal (Δ23–30%; p < 0.001; 1.00 ≤ d ≤ 1.63) compared with pubertal athletes (Δ14–26%; p < 0.05; 0.34 ≤ d ≤ 0.99). Due to the low number of pre-pubertal girls (n = 1–4), sex-specific differences were not computed for this cohort. Furthermore, significant sex by age interactions were found for anthropometrics, jump performance, and hand grip strength (p < 0.05; 0.27 ≤ d ≤ 0.45) (Table 1). Post-hoc analyses indicated that 12, 13, 14, and 15 years old male athletes showed significantly better CMJ performances compared with their female counterparts (9–24%; p < 0.05; 0.51 ≤ d ≤ 1.16). Furthermore, our analyses indicated that 14 and 15 year old males showed better DJ performance compared with females (i.e., DJ height, DJ performance index) (Δ11–24%; p < 0.05; 0.40 ≤ d ≤ 0.97). These sex-specific differences were even more pronounced with increasing chronological age. Sex-specific differences in body mass, standing and sitting height were only found in 15 years old athletes (boys > girls; Δ4–21%; p < 0.01; 0.56 ≤ d ≤ 0.94).

### Percentile values according to maturity status, age, and sex

Tables 3 and 4 illustrate sex-specific percentile values according to chronological age (Table 3) and maturity status (Table 4) for jump tests (CMJ, DJ), change-of direction speed tests (T-test), strength tests (Bourban-test, hand grip strength test), and balance tests (Y-balance test).

**Table 3:** Sex- and chronological age-specific anthropometric and physical fitness percentiles of German elite young athletes.

	age (years)	n	P_20_	P_40_	P_50_	P_60_	P_80_
**Anthropometry**							
**boys**							
standing height [cm]	12	54	150.6	159.0	160.8	164.0	170.4
	13	91	157.9	166.4	169.0	173.0	177.8
	14	68	162.4	166.4	169.0	171.7	180.1
	15	76	171.2	176.1	178.1	181.3	187.9
**girls**							
standing height [cm]	12	45	153.3	159.0	161.2	165.9	173.2
	13	59	160.1	164.3	165.5	167.0	171.3
	14	56	160.0	163.5	165.8	167.3	174.0
	15	52	165.5	169.0	170.9	172.5	176.2
**boys**							
sitting height [cm]	12	55	77.7	81.5	82.9	84.3	88.0
	13	90	81.0	85.0	87.5	88.1	91.6
	14	60	82.5	85.2	86.5	87.5	93.0
	15	67	87.2	90.9	92.0	93.2	95.9
**girls**							
sitting height [cm]	12	45	80.1	83.1	84.7	86.2	89.6
	13	58	83.7	86.0	87.5	88.0	90.1
	14	54	84.6	87.0	87.8	88.6	90.5
	15	51	86.6	89.1	90.0	90.4	91.7
**boys**							
BMI [kg/m^2^]	12	53	17.1	17.8	18.3	18.8	20.5
	13	88	17.2	18.4	18.8	19.5	21.3
	14	64	18.0	19.2	19.4	20.0	22.0
	15	68	18.7	20.3	21.4	22.1	24.0
**girls**							
BMI [kg/m^2^]	12	45	17.3	18.4	18.8	19.6	21.2
	13	58	18.3	19.4	19.9	20.3	21.8
	14	54	19.0	20.5	20.9	21.4	22.6
	15	49	19.1	20.5	21.0	21.6	23.0
**boys**							
body mass [kg]	12	53	39.5	45.1	48.2	49.8	59.7
	13	88	44.0	50.3	53.8	56.7	67.0
	14	64	48.5	54.5	55.5	60.0	68.1
	15	68	55.5	64.0	68.3	70.4	82.7
**girls**							
body mass [kg]	12	45	43.2	47.1	48.8	54.3	61.3
	13	60	49.1	52.8	54.4	55.8	62.1
	14	54	52.0	56.3	59.6	61.5	65.5
	15	49	54.7	58.7	61.6	63.7	69.1
**Physical fitness**							
**boys**							
countermovement jump [cm]	12	43	20.6	24.4	24.8	27.3	30.1
	13	86	24.4	26.3	27.4	28.4	30.2
	14	51	25.4	27.7	28.5	30.8	33.8
	15	61	29.9	32.6	34.4	36.9	41.1
**girls**							
countermovement jump [cm]	12	42	20.1	22.3	23.5	25.3	26.8
	13	53	21.4	23.6	25.3	26.5	28.5
	14	55	23.0	25.3	26.7	27.3	30.1
	15	47	23.0	25.0	26.4	27.7	31.6
**boys**							
drop jump (DJ) [cm]	12	43	18.0	20.6	21.9	23.1	25.4
	13	86	19.1	22.0	32.4	24.7	28.5
	14	51	21.8	24.5	25.8	27.5	31.0
	15	62	24.3	27.8	30.4	32.3	37.0
**girls**							
drop jump (DJ) [cm]	12	42	18.4	20.9	22.2	23.5	25.1
	13	52	19.1	21.2	23.0	24.1	26.0
	14	55	19.6	22.0	22.8	24.1	26.3
	15	49	19.8	23.8	25.3	26.3	28.5
**boys**							
drop jump ground contact time [ms]	12	42	290	236	217	209	189
	13	85	262	231	218	207	186
	14	47	259	219	206	202	188
	15	58	225	206	202	188	202
**girls**							
drop jump ground contact time [ms]	12	43	278	240	231	222	207
	13	50	248	221	208	200	177
	14	54	237	214	203	196	187
	15	49	233	215	206	196	187
**boys**							
drop jump performance index [m/s]	12	42	0.61	0.83	0.91	0.99	1.20
	13	86	0.81	1.01	1.04	1.10	1.28
	14	48	0.91	1.14	1.26	1.38	1.55
	15	58	1.18	1.41	1.52	1.65	2.03
**girls**							
drop jump performance index [m/s]	12	43	0.78	0.87	0.93	0.95	1.17
	13	51	0.85	0.97	1.04	1.15	1.37
	14	54	0.95	1.08	1.11	1.14	1.32
	15	49	0.89	1.05	1.20	1.27	1.44
**boys**							
T-test [s]	12	34	12.28	11.99	11.86	11.66	10.71
	13	75	11.96	11.21	10.93	10.76	10.30
	14	51	11.38	11.03	10.71	10.50	9.98
	15	62	10.67	10.22	9.99	9.89	9.64
**girls**							
T-test [s]	12	42	12.79	12.47	12.32	12.04	11.46
	13	52	12.41	12.10	11.84	11.66	10.76
	14	51	11.91	11.34	11.09	10.79	10.47
	15	48	11.65	10.83	10.71	10.53	10.20
**boys**							
Y-balance test (dominant) [%]	12	30	96.1	102.3	103.9	106.2	108.5
	13	36	95.8	97.5	101.1	104.2	108.6
	14	34	95.0	98.1	101.0	104.1	117.1
	15	45	96.9	100.1	104.4	107.1	112.6
**girls**							
Y-balance test (dominant) [%]	12	25	NA	NA	NA	NA	NA
	13	36	96.9	101.0	102.0	102.8	111.0
	14	36	99.0	104.7	105.0	106.8	108.4
	15	42	93.7	99.6	101.2	102.9	111.2
**boys**							
Bourban-test [s]	12	41	72	96	102	115	152
	13	80	79	100	123	150	192
	14	51	91	106	124	135	187
	15	61	77	105	119	125	148
**girls**							
Bourban-test [s]	12	42	57	75	83	95	142
	13	53	63	90	104	124	175
	14	52	71	88	99	109	158
	15	48	71	90	94	104	137
**boys**							
hand grip strength (dominant) [kg]	12	40	22.9	25.8	29.2	30.0	33.3
	13	74	25.1	29.4	30.8	33.5	40.7
	14	47	26.7	29.7	30.5	33.2	40.8
	15	48	30.9	37.0	40.1	44.8	51.7
**girls**							
hand grip strength (dominant) [kg]	12	40	22.6	25.3	26.4	27.5	30.1
	13	47	26.5	28.4	29.5	30.2	36.0
	14	39	27.3	31.0	32.7	33.7	36.0
	15	30	29.1	32.1	32.6	34.2	37.3

Sex-specific percentile reference values for anthropometric and physical fitness data. Data were only calculated if at least 30 participants were available within a subgroup. For the subgroups including 8, 9, 10, 11, 16, 17, and 18 year old athletes, less than 30 participants were available which is why these cells could not be filled.

NA = not applicable (< 30 participants).

**Table 4:** Sex- and maturity-specific anthropometric and physical fitness percentiles of German elite young athletes.

	maturity status	n	P_20_	P_40_	P_50_	P_60_	P_80_
**Anthropometry**							
**boys**							
standing height [cm]	pre-pubertal (mo: -1.97 ± 0.72)	77	141.1	149.1	152.0	154.8	159.6
	pubertal (mo: 0.04 ± 0.54)	162	159.5	165.2	168.0	169.9	175.7
	post-pubertal (mo: 2.44 ± 1.11)	149	174.8	180.2	183.7	186.5	190.8
**girls**							
standing height [cm]	pre-pubertal (mo: -2.18 ± 0.97)	3	NA	NA	NA	NA	NA
	pubertal (mo: 0.0 8 ± 0.58)	53	150.2	154.1	155.9	157.7	161.0
	post-pubertal (mo: 2.65 ± 1.04)	216	162.9	166.8	169.0	171.2	175.1
**boys**							
sitting height [cm]	pre-pubertal (mo: -1.98 ± 0.71)	78	73.5	76.9	78.3	79.6	82.2
	Pubertal (mo: 0.04 ± 0.54)	162	82.1	84.7	86.0	87.0	89.1
	post-pubertal (mo: 2.44 ± 1.11)	141	90.2	92.7	93.7	95.3	97.0
**girls**							
sitting height [cm]	pre-pubertal (mo: -2.01 ± 0.86)	4	NA	NA	NA	NA	NA
	Pubertal (mo: 0.06 ± 0.59)	54	77.4	80.7	81.5	82.6	85.8
	post-pubertal (mo: 2.65 ± 1.04)	212	85.3	87.8	88.7	89.5	91.5
**boys**							
BMI [kg/m^2^]	pre-pubertal (mo: -1.98 ± 0.72)	76	15.6	16.7	17.1	17.5	18.7
	pubertal (mo: 0.03 ± 0.54)	156	17.5	18.5	18.8	19.4	21.3
	post-pubertal (mo: 2.42 ± 1.14)	131	19.4	20.9	22.0	22.5	24.7
**girls**							
BMI [kg/m^2^]	pre-pubertal (mo: -2.18 ± 0.97)	3	NA	NA	NA	NA	NA
	pubertal (mo: 0.08 ± 0.58)	53	16.6	17.6	18.3	18.5	20.6
	post-pubertal (mo: 2.59 ± 1.00)	204	19.0	20.3	20.9	21.3	22.8
**boys**							
body mass [kg]	pre-pubertal (mo: -1.98 ± 0.72)	76	32.6	38.0	39.3	40.6	46.0
	pubertal (mo: 0.03 ± 0.54)	156	46.1	50.6	53.8	55.7	62.7
	post-pubertal (mo: 2.42 ± 1.11)	131	62.1	68.7	71.4	77.6	87.1
**girls**							
body mass [kg]	pre-pubertal (mo: -2.01 ± 0.86)	4	NA	NA	NA	NA	NA
	pubertal (mo: 0.06 ± 0.59)	54	37.4	44.0	45.2	47.1	52.4
	post-pubertal (mo: 2.60 ± 1.00)	204	52.3	58.0	60.0	62.6	68.7
**Physical fitness**							
**boys**							
countermovement jump [cm]	pre-pubertal (mo: -1.96 ± 0.74)	64	21.5	23.9	24.7	25.4	28.7
	pubertal (mo: 0.03 ± 0.55)	151	24.3	26.5	27.9	28.8	32.4
	post-pubertal (mo: 2.48 ± 1.05)	131	30.6	34.4	36.7	38.3	44.3
**girls**							
countermovement jump [cm]	pre-pubertal (mo: -1.64 ± NA)	1	NA	NA	NA	NA	NA
	pubertal (mo: 0.10 ± 0.56)	48	21.2	23.2	23.9	25.7	27.3
	post-pubertal (mo: 2.63 ± 1.03)	209	22.5	25.3	26.4	27.3	30.4
**boys**							
drop jump [cm]	pre-pubertal (mo: -1.96 ± 0.75)	63	18.0	19.7	21.0	21.9	24.5
	pubertal (mo: 0.03 ± 0.55)	151	19.6	22.7	23.7	25.0	29.2
	post-pubertal (mo: 2.54 ± 1.11)	135	24.9	28.7	30.4	32.4	37.0
**girls**							
drop jump [cm]	pre-pubertal (mo: -1.64 ± NA)	1	NA	NA	NA	NA	NA
	pubertal (mo: 0.10 ± 0.56)	48	18.4	20.9	22.6	23.7	25.6
	post-pubertal (mo: 2.63 ± 1.03)	209	19.6	22.7	24.1	25.1	28.0
**boys**							
drop jump ground contact time [ms]	pre-pubertal (mo: -1.98 ± 0.75)	62	262	202	195	190	177
	pubertal (mo: 0.03 ± 0.55)	144	269	226	216	208	189
	post-pubertal (mo: 2.55 ± 1.12)	128	229	206	200	191	180
**girls**							
drop jump ground contact time [ms]	pre-pubertal (mo: -1.64 ± NA)	1	NA	NA	NA	NA	NA
	pubertal (mo: 0.09 ± 0.55)	47	290	241	220	214	196
	post-pubertal (mo: 2.64 ± 1.03)	207	237	219	207	200	185
**boys**							
drop jump performance index [m/s]	pre-pubertal (mo: -1.98 ± 0.75)	62	0.76	0.97	1.00	1.10	1.30
	pubertal (mo: 0.03 ± 0.55)	145	0.80	1.00	1.10	1.16	1.41
	post-pubertal (mo: 2.55 ± 1.12)	130	1.18	1.38	1.47	1.63	1.98
**girls**							
drop jump performance index [m/s]	pre-pubertal (mo: -1.64 ± NA)	1	NA	NA	NA	NA	NA
	pubertal (mo: 0.09 ± 0.55)	47	0.74	0.85	0.90	1.04	1.19
	post-pubertal (mo: 2.64 ± 1.03)	209	0.90	1.04	1.12	1.21	1.42
**boys**							
T-test [s]	pre-pubertal (mo: -1.99 ± 0.76)	58	12.77	12.55	12.02	11.90	11.25
	pubertal (mo: 0.04 ± 0.55)	131	11.77	11.20	10.98	10.72	10.30
	post-pubertal (mo: 2.53 ± 1.10)	136	10.60	10.12	9.99	9.82	9.56
**girls**							
T-test [s]	pre-pubertal (mo: -1.64 ± NA)	1	NA	NA	NA	NA	NA
	pubertal (mo: 0.12 ± 0.54)	47	13.36	12.45	12.27	12.05	11.47
	post-pubertal (mo: 2.65 ± 1.04)	205	12.14	11.47	10.96	10.76	10.41
**boys**							
Y-balance test (dominant) [%]	pre-pubertal (mo: -1.74 ± 0.51)	39	97.7	102.1	102.7	105.3	108.1
	pubertal (mo: 0.10 ± 0.55)	64	94.3	97.7	101.1	106.9	111.7
	post-pubertal (mo: 2.61 ± 1.29)	88	97.6	101.7	103.6	106.6	116.0
**girls**							
Y-balance test (dominant) [%]	pre-pubertal (mo: -2.13 ± 1.01)	3	NA	NA	NA	NA	NA
	pubertal (mo: 0.01 ± 0.66)	16	NA	NA	NA	NA	NA
	post-pubertal (mo: 2.68 ± 1.00)	148	96.9	100.8	102.3	104.4	107.6
**boys**							
Bourban-test [s]	pre-pubertal (mo: -1.97 ± 0.76)	62	83	124	158	181	378
	pubertal (mo: 0.04 ± 0.55)	142	79	101	124	136	183
	post-pubertal (mo: 2.53 ± 1.11)	132	84	103	114	125	150
**girls**							
Bourban-test [s]	pre-pubertal (mo: -1.64 ± NA)	1	NA	NA	NA	NA	NA
	pubertal (mo: 0.10 ± 0.57)	47	75	101	126	139	184
	post-pubertal (mo: 2.64 ± 1.03)	202	68	87	98	110	148
**boys**							
hand grip strength (dominant) [kg]	pre-pubertal (mo: -1.96 ± 0.73)	55	19.4	22.1	23.4	23.9	26.5
	pubertal (mo: 0.03 ± 0.55)	130	25.6	29.5	30.7	32.5	39.0
	post-pubertal (mo: 2.30 ± 0.92)	99	33.7	41.3	44.9	47.6	52.9
**girls**							
hand grip strength (dominant) [kg]	pre-pubertal (mo: -1.64 ± NA)	1	NA	NA	NA	NA	NA
	pubertal (mo: 0.07 ± 0.59)	35	19.1	22.9	24.8	25.9	27.2
	post-pubertal (mo: 2.44 ± 0.95)	153	27.0	29.7	31.5	32.9	36.2

Maturity was determined by calculating the time from peak-height-velocity (PHV) according to the equation as provided by Mirwald et al., [21]. Maturity was classified as pre-pubertal (i.e., > 1 year before PHV), pubertal (i.e., ±1 year around PHV), and post-pubertal (i.e., > 1 year after PHV) [21].

mo = maturity offset; NA = not applicable (< 30 participants).

## Discussion

This study systematically aggregated anthropometric and physical fitness data of 703 elite young athletes aged 8–18 years from various sports and computed percentile values. Data were analyzed and expressed as percentile values according to maturity status, age, and sex. Findings indicate that anthropometry and physical fitness significantly increase with increasing maturity status and age, except for the Y-balance test. In general, male young athletes were taller, heavier, and they outperformed their female peers in CMJ, DJ, CoD, and hand grip strength performances. These sex-specific differences increase with increasing age.

### Maturity-, age-, and sex-specific differences in anthropometry and physical fitness

The pathway from childhood through adolescence into adulthood inevitably leads to body growth as well as somatic and cognitive maturation. Motor development depends on and is influenced by growth and maturation and consequently affects physical fitness [19]. Unlike chronological age, maturation is not a linear process. Skeletal, sexual and somatic maturation in children differ individually in timing and tempo which is why there is often a discrepancy between chronological age and maturation among youths [16–18, 34]. Therefore, maturity and chronological age should be considered when assessing anthropometry and particularly physical fitness in young athletes. Changes in individual structural constraints through growth are temporarily very dramatic. This is evident on a whole-body level (e.g., changes in size and proportion of the whole body) as well as on a system level (e.g., skeletal system, the muscular system, and the endocrine system) [35].

As hypothesized, our findings indicate that body height and mass significantly increase with increasing maturity status. Furthermore, post-pubertal athletes performed significantly better in various physical fitness tests (i.e., CMJ height, DJ height, DJ performance index, T-test, hand grip strength) compared to pubertal athletes. Thus, it seems that increases in body size, hormones, and muscle strength, caused by puberty, can improve physical fitness. There are rarely any studies that examined the effects of maturity on anthropometrics and physical fitness in children and adolescents. Jones et al. [36] reported that self-assessed stage of sexual maturity correlated positively with objectively measured physical fitness (i.e., vertical jump, 20-m shuttle run test, hand grip strength) in untrained boys and girls. Our results regarding the effects of biological maturity should be considered preliminary and appear to be in accordance with study findings from the general population of non-athletic youth [36].

In addition, our findings indicated that athletes were taller and heavier and they performed significantly better with increasing chronological age in selected physical fitness test (i.e., CMJ height, DJ height, DJ performance index, T-test, hand grip strength). Merely, physical fitness did not improve between 12 and 13 year old athletes as well as anthropometry did not change between 13 and 14 years old athletes. Data from different studies which have previously examined non-athletic youth [9, 19, 37] and athletic youth [13, 15, 38] confirm our findings in as much as improvements in physical fitness were reported with increasing chronological age. The performance enhancements can most likely be explained by changes in body size, physique, and body composition that are important factors affecting for physical fitness in general and muscle strength in particular [19, 39].

Furthermore, our findings indicate that young male athletes are taller as well as heavier compared to female young athletes. Further, results revealed that young male athletes outperformed young female athletes in the vertical jump, CoD- and hand grip strength test. Therefore, whilst stronger and leaner than many of their non-athletic peers, young female athletes are not as tall, as strong, nor as fast as their male counterparts [18]. The sex-specific anthropometric and physical performance differences are well in line with findings from previous studies regarding sexual dimorphism in the general population of non-athletic youth [16, 19, 20] and can most likely be attributed to higher absolute and relative strength levels in boys compared to girls [17, 18, 39]. The detected sex—maturity as well as sex—age interactions indicated that differences in physical fitness outcomes increased between male and female young athletes with increasing maturity and chronological age respectively. Previous studies highlighted that sex differences in physical fitness are rather small prior to the onset of puberty [19]. This finding could not be demonstrated in our results, due to a small sample size in pre-pubertal children, especially in girls. However, during the adolescent growth spurt, sex differences become more pronounced [15]. This can mainly be explained by hormone-dependent changes in body composition [15, 19, 39]. Boys show significant rise in growth of bone, stature and muscle mass and simultaneous loss of fat in limbs under the influence of testosterone [17, 39]. Moreover, results of several studies indicate that testosterone is responsible for improved anaerobic enzyme systems and structural development of fast twitch muscle fibers in muscles [40]. Thus, an increase in testosterone may determine the greater formation and development of fast twitch muscle fibers that positively affect the performance of explosive muscle actions [40]. Girls experience lesser increment in stature and muscle mass, but a significant accumulation of body fat [18, 39]. Thus, the beneficial effects of maturational changes are present but less evident in girls where sport-related motor performances tend to plateau from mid-adolescence. Thus, as a result of sex-related differences in growth and maturation during adolescence, the post-pubertal male athlete is stronger and has more muscle mass than the post-pubertal female athlete [35].

### Physical fitness percentiles

Chronological age provides a useful point of reference when referring to growth and maturity status. However, biological processes do not progress in a linear fashion [17–19]. Therefore, youth of the same chronological age display wide variability in the development of morphological and physiological characteristics. This is a major challenge in youth sport where competitions mainly are regulated by chronological age-groups to establish equal chances of success for all athletes [16, 41]. But, within a prescribed age-group, variations in maturity status can deliver a distinct advantage not only in performance but also for talent identification. For example, boys who mature earlier are generally taller, heavier, have higher mass-to-stature ratios and, thus, are generally more prone to success in most types of exercise, particularly in those that involve strength, velocity and power [16, 17] than those who mature at a later age. Many young athletes drop out of sport or are cut from sport squads for instance due to retarded timing and tempo of their growth and maturation. For this reason, practitioners and coaches should be aware of the effects of age, growth and maturation on sports performance and should provide opportunities for all talented children irrespective of the maturational status [16, 17]. Maturity-specific reference for anthropometry and physical fitness tests for male and female young athletes are necessity to assess a youth athletes’ performance adequately. Due to lack of literature that examines maturity-specific anthropometric and physical fitness percentiles for male and female youth, especially young athletes, findings are preliminary.

In terms of the established chronological age-specific percentile reference values of anthropometry and physical fitness tests for male and female young athletes, previous research mainly examined the general population of untrained children and adolescents. For juvenile non-athletes, studies with large cohorts are available [7, 9]. For instance, Tomkinson et al. [9] established sex- and age-specific percentile reference values for physical fitness (e.g., hand grip strength, bent-arm hang, standing long jump, 20-m shuttle run) in children and adolescents aged 9–17 years. However, young athletes represent a small segment of the general population with regards to their motor skill and performance levels. This is due to their genetic predisposition but also their exposure to regular training [11, 42]. Accordingly, anthropometric and physical fitness norms from the general youth population cannot be translated to young athletes [42]. Only few studies are available that provide age- and sex specific mean values for youth from different sports [13–15, 38]. However, given that none of them established age- and sex-specific percentiles, findings are again preliminary.

### Study limitations

We enrolled a convenience sample in this study consisting of 703 male and female young elite athletes from 18 different sports. The distribution of age and sex of the tested elite young athletes is not the same for each different sport. Due to the variety of sports within each sub-group the results may be affected by variations in morphology, growth, maturity status, and/or physique among athletes of different sports. Furthermore, the sample size of each sub-group is rather small and varies according to the sub-group under investigation (e.g., pre-pubertal girls are under-represented). Doing research in elite (youth) sports is always limited as to the size of the available cohort. The overall sample of young athletes is small compared to the general youth population. Consequently, it is not appropriate to compare the size of our study cohort with previous studies reporting norm values of the general youth population. In this study, we were able to enroll 703 male and female elite young athletes aged 8–18 years. While we acknowledge that the included number of participants is small when compared to studies using the general youth population, it is rather large compared to other studies who examined youth athletes [13, 43, 44]. Nonetheless, due to the small sample size in several sub-groups, the established reference values have to be interpreted with caution. Furthermore, we acknowledge that different individuals measured and tested the enrolled athletes. However, test instructions and test protocols were highly standardized and the observers were experienced exercise scientists. Moreover, we have applied the predicted maturity offset method according to Mirwald et al. [21] to estimate participants’ maturity status. The application of the gold standard (x-ray exams of the left wrist) would have certainly provided higher accuracy according to the actual maturity status. However, this method is costly and causes radiation exposure which is why we decided to apply the Mirwald method based on anthropometrics (i.e., sitting and standing height). Müller et al. [45] performed a cross-validation study with young athletes using x-ray exams and the Mirwald method and concluded that the prediction equations to determine age at PHV appears to be a valid method for the assessment of biological maturity. Finally, the applicability of our observations to young athletes of other countries and races may be limited.

## Conclusions

This study examined maturity-, age-, and sex-specific differences in anthropometrics and physical fitness in young athletes from various sports. Our findings indicate that body height and mass increased significantly with increasing maturity status and chronological age. Further, our results showed that physical fitness (i.e., CMJ height, DJ height, DJ performance index, DJ ground contact time, T-test, hand grip strength) was significantly better in post-pubertal compared to pubertal athletes. In addition, physical fitness outcomes (i.e., CMJ height, DJ height, DJ performance index, T-test, hand grip strength) improved with increasing chronological age (i.e., 12 = 13 < 14 < 15 years).

Furthermore, maturity-, age- and sex-specific percentile reference values including a wide range of physical fitness outcomes (i.e., CMJ, DJ height, DJ performance index, DJ ground contact time, CoD speed, dynamic balance, trunk strength endurance, hand grip strength) were established. The percentile reference values add value to existing norms for children and adolescents for the specific sub-population of trained youth. Practitioners and coaches can use the established percentile values as approximate benchmarks to identify and develop young athletes with specific fitness characteristics for talent identification and development.

## Supporting information

S1 Data (XLSX)
